# Prevalence, Risk, and Challenges of Extemporaneous Preparation for Pediatric Patients in Developing Nations: A Review

**DOI:** 10.3390/pharmaceutics15030840

**Published:** 2023-03-04

**Authors:** Sri Hartati Yuliani, Dina Christin Ayuning Putri, Dita Maria Virginia, Michael Raharja Gani, Florentinus Dika Octa Riswanto

**Affiliations:** 1 Division of Pharmaceutics and Pharmaceutical Technology, Faculty of Pharmacy, Universitas Sanata Dharma, Yogyakarta 55282, Indonesia; 2 Division of Pharmacology and Clinical Pharmacy, Faculty of Pharmacy, Universitas Sanata Dharma, Yogyakarta 55282, Indonesia; 3 Division of Pharmaceutical Analysis and Medicinal Chemistry, Faculty of Pharmacy, Universitas Sanata Dharma, Yogyakarta 55282, Indonesia

**Keywords:** extemporaneous preparation, pediatric, developing nations

## Abstract

Extemporaneous preparations are still widely prescribed for pediatric patients with special treatments of certain doses and/or combinations of drugs. Several problems related to extemporaneous preparations have been linked to the incidence of adverse events or a lack of therapeutic effectiveness. Developing nations are facing the challenges of compounding practices. The prevalence of compounded medication in developing nations must be explored to determine the urgency of compounding practices. Furthermore, the risks and challenges are described and explained through investigation and collection of numerous scientific articles from reputable databases, including Web of Science, Scopus, and PubMed. Pediatric patients need compounded medication related to the appropriate dosage form and dosage adjustment. Notably, it is important to observe extemporaneous preparations in order to provide patient-oriented medication.

## 1. Introduction

Compounding is the art and science of making a special preparation (customized) to meet the special needs of patients or compensate for the unavailability of commercial preparations [1]. Compounding is an important activity of pharmacists that aims to provide dosage forms or certain doses of unavailable medicine among licensed drugs [2]. Extemporaneous preparations remain widely used, especially for pediatric patients [3,4], patients with rare diseases [5,6], patients who require special dosage forms [7] or certain doses/strengths, and dermatological patients [8,9]. Compounding is performed by various pharmaceutical services, including in pharmacies, hospitals, and health centers [3,10].

The prescription of extemporaneous preparations (including unlicensed and off-label drugs) is a common practice among pediatricians [11]. Some extemporaneous preparations are associated with the incidence of adverse events in pediatric patients [12]. The use of licensed drugs for pediatric patients has the potential to be dangerous, because excipients are not suitable for children, even if administered in small amounts [13]. Since extemporaneous compounding is described as a branch of pharmacy practice aiming to produce appropriate pharmaceutical preparations when there are no commercially available, licensed, and age-specific dosage forms, problems related to stability, pharmacokinetic profile, and drug effect may potentially occur [9].

Developing nations encounter population growth, which is followed by an increase in the pediatric population. In addition, the prevalence of compounding practice in developing nations indicates the urgency of establishing guidance from the government for compounding practices. Regarding the frequent compounding practices in developing nations, the risks of extemporaneous preparations should be noted in order to reduce adverse drug reactions (ADRs) and elevate the therapeutic effect [14,15]. It was confirmed that the incidence of ADRs in infants is two-fold higher because the infant’s body is not physiologically mature [11]. The safety and efficacy of extemporaneous compound preparations used in health services to fulfill the special needs of patients are very dependent on the professionalism of the pharmacist [5].

The challenges of compounding practices should be detailed to allow for the identification of the problems with those procedures. Previous studies have observed several challenges, especially regarding stability and incompatibility, professional knowledge and skill, and regulatory guidance [13,16,17,18]. In addition, information regarding the stability of compound preparations is still limited [17]. Thus, those challenges could endanger patients and cause problems related to ADR and effectiveness. Therefore, pharmacists must control stability and compatibility and provide formulation information to ensure patients are supplied with safe, high-quality, and effective preparations [19]. The current narrative review aims to present integrated information on pharmaceutical compounding products’ prevalence, risks, and challenges related to pediatric patients in developing nations.

## 2. Methods

This narrative review article was prepared by identifying, investigating, and collecting numerous original research articles, review articles, and related books from reputable databases, including Web of Science, Scopus, and PubMed. A literature investigation was conducted between July and September 2022. The keywords explored during the literature search were “quality assessment of compounding”, “stability of compounding”, “compatibility drugs”, “extemporaneous preparation”, “pediatrics”, and “compounding safety”.

## 3. Results and Discussion

### 3.1. The Prevalence of Compounded Medications for Patients in Developing Nations

The living standards, incomes, and economic and industrial development in developing nations were lower than the average international conditions. According to the International Monetary Fund (IMF) definition, there are 152 developing nations with a current population of about 6.69 billion, which is a significant percentage (85.33%) of the world’s population. It covers all of Central and South America, all of Africa, most of Asia, and many other island nations [20].

The prevalence of extemporaneous preparation usage in Indonesia, one of the developing nations, is less than 5% of all prescriptions [21]. Pediatric patients receive more compounded preparations than adult patients [22]. The prevalence of children’s extemporaneous preparations is high in Indonesia. As many as 71.5% of the drugs administered to children are compound preparations [23]. From primary health services in Indonesia, pediatric patients aged 0–5 years (74.33%) mostly receive extemporaneous preparations, the majority of which are divided powders (88.36%) and oral suspensions (8.06%) [24]. In Thailand, 73.5% of pediatric patients receive off-label drugs [25]. In Brazil, newborns admitted to the neonatal care unit (NCU) also receive compound preparations [26]. According to Koszma et al., 98.1% of newborns hospitalized received at least one off-label drug [27]. In Estonia, 97% of hospitalized newborns receive two extemporaneous preparation products on average [28]. In addition to pediatricians, dermatologists also prescribe many extemporaneous preparations [29].

Table 1 describes the compounding reports related to non-sterile extemporaneous products and their proportions in developing nations. The compounding process tends to be performed in pharmaceutical practice in developing nations. Oral and semisolid dosage forms are predominantly prescribed as extemporaneous products. These require standard guidelines and procedures such as the quality control of extemporaneous products.

Compounding practices are still carried out in developing nations. The quality of the extemporaneous preparation products was assessed by evaluating their physical, chemical, microbiological, and therapeutic profiles. A study showed that gabapentin oral suspension preparations have good physical, chemical, and therapeutic qualities after being made; however, after storage for up to 3 months, a cake formed in the suspension, the drug levels were decreased, and the anticonvulsant activity was reduced [33]

The quality of extemporaneous preparations, especially those for dermatology patients, has to be considered. Several compounded preparations may lead to unpredictable effectiveness and potential toxicity [34]. Microbial contamination should be considered since it could be harmful for the patient. Several active ingredients are used as customized compounds for pediatric dermatology therapy, such as triamcinolone, gentamycin, hydrocortisone, permethrin, salicylic acid, etc. [29]. The research of Hapsari et al. reported that a combination preparation of gentamicin ointment and hydrocortisone cream used in three primary health care centers was contaminated with *Pseudomonas aeruginosa, Salmonella* spp., *Staphylococcus aureus*, and *Escherichia coli* [35].

On the other hand, other previous studies presented good evaluation results for extemporaneous preparation products. An extemporaneous preparation containing voriconazole in two types of vehicles showed good stability over 30 days of storage both at room temperature and under refrigeration [36]. Research by Gani et al. showed that an oral divided powder mixture containing ambroxol and salbutamol had levels in accordance with compendial requirements and could maintain physical and chemical stability for up to 7 days of storage [37].

### 3.2. Potential Risks of Pharmaceutical Compounding

In contrast to licensed drugs, extemporaneous preparations do not undergo a clinical evaluation process for their efficacy and safety. Extemporaneous preparations made for small groups of patients risk causing adverse events. The risks of drug compounding include formula failure, microbial contamination, calculation errors, patient acceptance issues, health and safety risks, therapeutic risks, clinical consequences, and factors related to clinical risk [18]. During the drug compounding process, grinding, diluting, adding additional ingredients, etc., errors that affect the efficacy and safety as well as patient acceptance, can occur [25]. The previous study observed variations in the extemporaneous glucocorticoid for asthma therapy in children. It was confirmed to affect the length of hospitalization and other adverse effects (12 cases found) [38]. Therefore, it is necessary to establish standards for the compounding process, including standard formulas, appropriate equipment, and trained personnel [39].

In certain situations, the use of extemporaneous preparations in children is unavoidable, especially in situations where there is no alternative treatment, with the consideration that the benefits outweigh the risks [23]. However, patients are at risk because the efficacy and safety of the extemporaneous preparations cannot be ascertained [40]. Excipients are considered materials that are inert and do not have pharmacological activity, so they are considered safe for patients. However, children’s responses to drugs may differ from those of adults. Infants are the most vulnerable group due to their immature organs and differences in the pharmacokinetics and pharmacodynamics of drugs in their bodies. Therefore, excipients that are safe for adults are not necessarily safe for infants, even though drugs for adults are often formulated for this population [41]. In a study in Japan by Saito et al., there were 343 infants admitted to the NICU. They received 2360 prescriptions for 426 products containing 228 active pharmaceutical ingredients (APIs). Excipients of concern were found in 52 (12.2%) products in 646 (27.4%) prescriptions for 282 (82.2%) infants [42]. Pediatric preparations should use minimal amounts of excipients to ensure that the resulting preparations meet the criteria for stability, palatability, patient acceptance, and uniformity of dosage [43]. Table 2 presents previous studies which observed potential excipients of concern related to pediatric preparations.

Several literature reviews have summarized the safety and toxicity of pharmaceutical excipients for pediatric patients [44,45,46,47,48]. Health professionals should increase their awareness of the risk of adverse events brought on by excipients. Some excipients, such as arginine, aspartame, paraben, polyethylene glycol, polysorbate, sorbitol, ethanol, etc., have been reported to cause an adverse reaction. Parabens were the highest reported excipients and were linked with several cases of adverse reactions in the pediatric population. Further, parabens were found to be associated with hypersensitivity reactions in the pediatric population, especially in neonates [49]. Another study reported the risk of medication with sorbitol as an excipient with potential adverse effects, such as diarrhea. Thus, it is important to consider the medication of pediatric and critical disease patients. According to the European excipient review, the daily admissible intake of sorbitol is limited to 5 mg/kg in children 0–2 years old and 140 mg/kg in those older than two years [50].

A study screening 2155 compounding reports identified 21 concentration errors and 27 contamination errors. The errors caused harm to 1119 patients. Errors in concentration largely affect children, whereas those in contamination are predominantly an issue in parenteral use [7]. Concentration errors should be of concern, considering their potential to lead to overdoses or underdoses, especially in special populations, having the potential to affect the safety and efficacy of extemporaneous products [51,52,53]. Therefore, information on the patient’s age and disease is required in the consideration of the recommended dose, especially for an extemporaneous product [54].

### 3.3. The Challenges of Compounding Practices

Several studies have reported the challenges in compounding practices in developing nations, including a lack of competence among staff, a lack of systematic electronic documentation, insufficient facilities and funding, a lack of inspection and quality control, the absence of national formula guidelines for extemporaneous preparations, and a lack of information on the age-appropriateness of dosage forms [34,54,55,56]. Further, specific data related to the quality of extemporaneous products need to be collected to guide pharmacists. A previous study reported that pharmacists tended to not assess their compounding products because they did not have specific standards. Consequently, pharmacists who practiced compounding only evaluated their products by conventional methods [57]. A preventive action that could be implemented is for pharmacists to always prepare fresh products to reduce the risk of degradation or contamination with microorganisms. The products have relatively short shelf-lives [58]. In brief, this study focused on factors that present challenges in compounding practices, such as stability and compatibility issues, the need for professional training, and a lack of regulatory guidance.

**Table 2:** Excipients of concern for children.

Class of Excipient	Excipient	Negative Effect for Children	References
Preservative	Parabens	Parabens may cause hyperbilirubinemia in neonates when administered as an injectable preparation.	[44]
	Sodium benzoate	Sodium benzoate in an injectable form can cause atopic dermatitis, urticaria, and anaphylactic shock.	[41,43,44]
	Benzalkonium chloride	Benzalkonium chloride can cause dose-related bronchoconstriction in pediatric asthma patients. It also induces ototoxicity, irritation, and hypersensitivity.	[28,45]
	Benzyl alcohol	Benzyl alcohol leads to intraventricular hemorrhage and metabolic acidosis in neonates when administered intravascularly and intramuscularly.	[46]
Solvent	Ethanol	Ethanol may suppress the central nervous system, especially for children younger than 6 years old.	[59]
	Glycerol	A glycerol concentration of more than 40% may cause several side effects in children, e.g., diarrhea, headache, electrolyte disturbances, and stomach upset.	[44]
	Propylene glycol	A high dose of propylene glycol can cause lactic acidosis, central nervous system depression, coma, hypoglycemia, seizures, and hemolysis.	[46,60]
	Polyethylene glycol	Polyethylene glycol 3350 may lead to abdominal pain, diarrhea, and vomiting when used for constipation treatment in children.	[47]
Antioxidant	Sulfites	The potential side effects of using sulfites in children are dermatitis, diarrhea urticaria, asthma, anaphylactic reactions, and hypotension.	[48]
	Propyl gallate	The potential side effects of propyl gallate are dermatitis, skin allergies, and methemoglobinemia.	[46]
Sweetener	Saccharin sodium	The potential adverse effects of sodium saccharin are urticaria with pruritus and photosensitivity reactions.	[28,61]
	Sucralose	The potential negative effects of sucralose are carcinogenicity and diabetes.	[62]
	Sorbitol	The potential adverse effects of sorbitol are diarrhea, liver damage, flatulence, and abdominal pain.	[44,48]
	Peppermint	Peppermint may cause a burning sensation, atrial fibrillation, and muscle pain.	[63]
	Aspartame	Aspartame is associated with an increased risk of type 2 diabetes, cardiovascular diseases, nonalcoholic fatty liver disease, hormone-related cancers, and an elevated risk of early menarche (among girls aged 9–10 years).	[64]
Filler	Lactose	The potential negative effects of lactose are metabolic acidosis, eczema, dehydration, flatulence, diarrhea, join pain, and abdominal pain.	[65]
	Mannitol	A potential adverse effect of mannitol is diarrhea.	[66]
Coloring agent	Tartrazine	Tartrazine (yellow number 5) has been implicated in anaphylactic reactions, edema, asthma, bronchospasm, eosinophils, angioedema, and hives in patients with sensitivity to it.	[46]
	Azo dyes	Azo dyes may induce hyperactivity behavior and chromosomal aberrations.	[67]

#### 3.3.1. Stability and Incompatibility Related to Quality Assessment of Extemporaneous Preparation

In compounding practice, stability and compatibility are among the important factors determining the product’s quality, efficacy, and safety [24,68,69,70]. However, the disruption of the original packaging’s integrity causes changes in the chemical, physical, microbiological, pharmacological, and toxicological stability of extemporaneous drug formulations. The information and literature about the stability, storage conditions, packaging, and handling techniques for the preparation of extemporaneous formulations are also limited [71,72]. Stability is defined as the extent to which a product retains, within specified limits, and throughout its period of storage and use (i.e., its shelf-life), the same properties and characteristics that it possessed at the time of its manufacture [73,74].

Stability can be classified as chemical, physical, therapeutic, toxicological, and microbiological stability. Chemical stability can be defined as the retention of each active ingredient’s chemical integrity and potency, within the specified limits. The study of a compounded powder containing ambroxol hydrochloride and salbutamol sulfate indicated the occurrence of drug degradation. However, the drug content still met the compendial requirements after seven days of storage [20]. Physical stability is related to the original physical properties, including appearance, palatability, uniformity, dissolution, and suspension ability. Several studies have shown incompatibility between active substances and excipients. Acetylsalicylic acid is incompatible with polyvinylpyrolidone K30 (PVP) and magnesium stearate due to a possible physical interaction with colloidal silicon dioxide and stearic acid [75]. Acetylsalicylic acid is known as an analgesic used for pediatrics [76]. However, several countries in the European Union are permitted to offer acetylsalicylic acid prescription free [77] with certain active ingredients. The physical stability of an amlodipine and omeprazole suspension was assessed, showing that, when the suspension was left undisturbed for a long period of time, the particles aggregated, sedimented, and eventually caked [19].

A lack of change in the therapeutic effect is related to the stability of a therapeutic. The toxicological stability can be associated with the minimum toxicity effect. The sterility or resistance toward microbial growth according to the specified requirements can be evaluated to assess the microbiological stability [13,78,79,80]. A previous study found that the risk of contamination was increased 100-fold in extemporaneous products compounded by nurses or other medical staff compared to those prepared by pharmacists [81]. Pharmacists should consider both microbial and material contaminations, which pose a danger to patients [82,83,84]. Good aseptic procedures are required to prevent microbial contamination, and appropriate techniques are needed to avoid material contamination [85]. In addition, antimicrobial agents are required to retain effectiveness within the specified limits. The microbial stability of an extemporaneous preparation can be improved by adding preservatives. The microbial stability of an oral suspension containing sildenafil sulfate and losartan could be maintained by using a preservative in the formulation [21,22].

Many factors may affect the stability of drug substances and dosage forms, but the following can be considered two major factors: First, the environmental conditions under which the active ingredient or excipient is compounded, packaged, or stored [80], and second, the actual dosage form or active ingredient. Furthermore, the capability, knowledge, and responsibility of the pharmacist become key to the maintenance of the stability of the active ingredient [86,87].

Environmental factors can affect the stability of active compounds, such as temperature, light, humidity, radiation, air, particle size, solvents, pH, and the presence of other chemicals resulting from contamination or from the intentional mixing of different products, as well as the cleaning and sterility of the room [80,88]. The dosage form affects the chemical reactions between the API and environment or compatibility of the API and excipient. There is usually no obvious visual or olfactory evidence of their occurrence [89].

The stability or shelf-life of compounded products is typically not verified by stability testing [80]. Therefore, compounded preparations cannot be assumed to retain their original strength and purity over time [90]. Consequently, they are marked with beyond-use dates (BUDs) as opposed to expiry dates. The BUD is defined as the date or time after which a compounded sterile preparation (CSP) or compounded nonsterile preparation (CNSP) may not be stored or transported and is calculated from the date or time of compounding [91,92,93].

Incompatibility is defined as an undesirable change or an undesirable product being formed upon the mixing of components, which may affect the safety, efficacy, appearance, and stability of the pharmaceutical product [94]. It can occur at any point throughout the supply chain of a drug (from compounding/manufacturing until storage). Incompatibility may occur between an API and API or API and excipient, and may be detected by changes in the physical, chemical, and therapeutic qualities of medicines [95].

Physical incompatibility can be detected by visual inspection, such as the detection of a color change or the formation of a precipitate or crystal. It can be mediated by chemical reactions such as acid–base reactions, complexation, or the formation of poorly soluble salts or heavy metals [78]. Chemical incompatibility cannot be detected by visual inspection. It can be detected by using instruments such as High Performance Liquid Chromatography (HPLC) or Liquid Chromatography–Mass Spectrometry (LC–MS) apparatus [96]. This is because chemical incompatibility can lead to the degradation of substances by chemical reactions such as oxidation–reduction that are irreversible and impossible to detect visually [97]. Therapeutic incompatibilities occur for reasons including the following: Errors in dosage, wrong doses or dosage forms, contraindicated drugs, synergistic and antagonistic drugs, and drug interactions [98].

There are some factors influencing physicochemical incompatibility reactions, such as the concentration, the pH, and solvents. Solutions with higher concentrations are potentially unstable. Regarding pH, it is important to avoid acid–base incompatibility; for example, combining adrenaline (pH = 3) and acyclovir (pH = 11) may cause problems. Regarding the solvents used to dissolve APIs, differences in pH or polarity can make them incompatible [98].

#### 3.3.2. Professional Training

A compounding pharmacist must have access to high-quality compounding supplies and utilize their professional knowledge to design novel formulae and dosage forms to satisfy patients’ individual needs. A compounding pharmacist can provide a vital service in treating pediatric patients if they have the necessary skills, knowledge, competence, and resources [99]. All staff involved in extemporaneous preparations must receive training appropriate for their role. Typically, this will (a) provide them with knowledge about good extemporaneous preparation practice, local practices including health and safety issues, formulations, expiry periods, quality assurance appropriate for the level of involvement, risk assessment, the potential for medication errors, the pharmacy, and the products and services provided by the pharmacy, and (b) equip them with proficiency in the required extemporaneous preparation abilities, as well as in pharmaceutical calculations and dilutions. Staff must be closely supervised and verified during training, and learners must understand the boundaries of their responsibility. When contemplating delivering extra compounding services in an institution, pharmacists should not expect this to significantly alter their practice in terms of the time needed for compounding. A defined training program should be accessible, and the completion of training should be documented. This applies to all preparation professionals, including those who are not actively involved in preparation activities (e.g., cleaning staff) [5].

A lack of awareness and knowledge among compounding staff should be considered a risk factor that influences pharmaceutical compounding products. The outbreak in 2012 raised awareness of the need for the careful selection of pharmacists in compounding areas to ensure that the compounding process is performed in appropriate circumstances [18]. In fact, compounding is one of the competencies of a pharmacist [21]. Several studies have mentioned that pharmacists have higher awareness and knowledge of compounding than other healthcare workers [86,87,89], thus reducing the risk in the compounding process.

Fent et al., in their research, found that lisinopril (used to treat high blood pressure), levothyroxine (used to treat hypothyroidism), and methotrexate (a drug prescribed for cancer) were detected in the air of a compounding room. The compounding process also has the potential to endanger the dispensing personnel due to exposure to dust from the drug-grinding process [100]. The study demonstrated that professional training is required for extemporaneous preparations.

#### 3.3.3. Lack of Regulatory Guidance

Extemporaneous preparations have never been monitored by regulators, formulators, or even policymakers in health services [91]. Extemporaneous preparations are not required to meet certain standards from the regulator [92]. The main purpose of regulation is to ensure the efficacy and safety of drug use [3].

Several developed countries already have guidelines for the compounding of non-sterile drugs. It is notable that Canada has established complete regulatory compounding guidance. In Canada, there is a “Guidance Document for Pharmacy Compounding of Non-sterile Preparations”. The guidance aims to provide pharmacists and pharmacy technicians who compound non-sterile preparations with the details needed to evaluate their practices, develop service-related procedures, and implement appropriate quality control for both patients and compounding personnel, with a view to guarantee the overall quality and safety of non-sterile preparations. However, several cases have been reported in Canada through ISMP. A case related to a compounding failure that resulted in the death of a pediatric patient occurred. Compounding errors can include the inaccurate measurement of the active substance formulated for the patient. The regulatory guidance could facilitate the evaluation of compounding cases. Ensuring compliance with the compounding guidelines could be an approach to preventing compounding errors, especially regarding risk management [93,101].

The standardization of extemporaneous products and examination of product quality should be considered in compounding [2]. Establishing standards for extemporaneous products prompts pharmacists to pay attention in the compounding process, including in the selection of raw materials, risk assessment, procedures, and quality of the products. Moreover, it is urgent for the government to establish specific guidelines for extemporaneous products to guarantee that the products are suitable and safe for patients [44,102].

## 4. Conclusions

Compounding practices for pediatrics are widely applied in developing nations. The pharmacokinetics and pharmacodynamics of drugs for pediatric patients differ from those for adults. Therefore, the efficacy and safety of extemporaneous preparations for pediatric patients should be considered. However, several studies found potential risks of compounding practices, including calculation error, microbial contamination, dose uniformity, improper excipients, and patient acceptance. Compounding practices encounter various challenges, including stability and incompatibility of extemporaneous preparations, lack of competence among compounding staff, and the absence of national guidelines for compounding practices.

## Figures and Tables

**Table 1:** Compounding reports related to non-sterile extemporaneous products and their proportions in developing nations.

Studies	Country	Population/ Patients	Dosage Form	Finding	References
Compounding Practice in a Developing Country: A Case Study of Divided Powder in Indonesia	Indonesia	666 prescriptions	Divided powders	100% oral divided powder	[9]
The Extemporaneous Compounding at Primary Health Care Centers: Characteristics and Personnel	Indonesia	1200 patients	Oral powder, suspension, semisolid dosage form	1229 final dosage forms: 88.36% oral powder, 8.06% suspension, and 3.58% semisolid dosage form	[24]
Profile and Determinants of Compounding Services among Pharmacists in Indonesia	Indonesia	780 prescriptions	Powder, capsule, cream, syrup	Powder, 32.05%; capsule, 25.26%; syrup, 21.92%; and cream, 20.77%	[30]
Prevalence, determinants, and characteristics of extemporaneous compounding in Jordanian pharmacies	Jordan	223 prescriptions	Tablets, capsules, suppository, ointment, cream, solution, suspension, syrup, paste powder	Cream, 99.6%	[2]
Evaluation of extemporaneous compounding in tertiary hospital pharmacy in the Polokwane Municipality: A pilot study	South Africa	691 batch records	Cream, ointment, solution	Cream, 33.0%; ointment, 13.60%; solution, 43.9%	[6]
Prescribing Pattern of Dermatological Compounding in Ethiopia: The Case of Alert Hospital	Ethiopia	441 prescriptions	Cream, ointment, solution, lotions	464 final products: ointment, 53%; cream, 40.1%; solution, 5.8%; lotion, 1.1%	[31]
An Overview of Pharmaceutical Production in Thai Hospitals	Thailand	1347 hospitals	Solid, liquid, semisolid	Solid, liquid, and semisolid products in the general hospital were 35.29%, 36.21%, and 39.13%, respectively.	[32]

## Data Availability

Not applicable.
